# Pregabalin addiction caracteristics at oran: A cohort study

**DOI:** 10.1192/j.eurpsy.2021.1491

**Published:** 2021-08-13

**Authors:** C. Yasmine, D. Aicha

**Affiliations:** Addictology Service, specialized hospital establishment in psychiatry sid chami, oran, Algeria

**Keywords:** addicion, pregabalin, misuse, abuse

## Abstract

**Introduction:**

Pregabalin is an analogue of gamma-aminobutyric acid (GABA). Recent reports suggest illicit pregabalin use may be increasing among youth, however its addictive potential has not been well established (1).

**Objectives:**

Drug seeking behavior and chronic drug use are associated with defcits in glutamate clearance and activation of postsynaptic glutamatergic receptors (2). Based upon multiple studies, we compare here the addiction and misuse risks of pregabalin with those of traditional psychoactive substances (3).

**Methods:**

Users of pregabalin were identified from 1st January 2019 to 31 December 2019 in Oran Addictology service, at west Algeria. The aim of the study was to establish the addictive potential of pregabalin and to compare the addiction risks of pregabalin with traditional psychoactive substances in west Algerian population. Clinical diagnosis was established according DSM-5 diagnosis criteria.

**Results:**

A total of 92 cases of pregabalin abuse or dependence were identified. The principal population at risk consists of patients with other current or past substance use disorders, for the most part opioid and multi-drug users, the age group were between 17-38 years old, mostly single men. The mean daily dose of pregabalin was 1200 mg. Almost all patients experienced withdrawal symptoms when pregabalin was discontinued.

**Conclusions:**

The misuse of pregabalin often leads to abuse and dependence, mostly in the context of multiple drug addiction, especially in youth population.

